# Facilitators and barriers to community engagement in the Global Polio Eradication Initiative–A mixed methods study

**DOI:** 10.1371/journal.pgph.0001643

**Published:** 2023-04-07

**Authors:** Priyanka Agrawal, Abigail Neel, Assefa Seme Deresse, Sue Gerber, Olakunle Alonge

**Affiliations:** 1 Department of International Health, Johns Hopkins Bloomberg School of Public Health, Baltimore, Maryland, United States of America; 2 School of Public Health, College of Health Sciences, Addis Ababa University, Addis Ababa, Ethiopia; 3 Global Development, The Bill and Melinda Gates Foundation, Washington, United States of America; University of Washington Bothell, UNITED STATES

## Abstract

Community engagement (CE) is an important component of public health research and program implementation, especially in low- and middle-income countries. More recently, CE activities have been utilized to develop partnerships in research and program implementation processes, and advocate for policy recommendations with the aim to improve acceptance and reduce disparities of public health research activities and benefits in the involved communities. Utilizing the tacit knowledge gained from the Global Polio Eradication Initiative, this paper highlights the contributors and challenges to the implementation of the GPEI program’s community engagement initiatives from an implementers’ perspective. The study took a mixed methods approach to analyze data collected from the Synthesis and Translation of Research and Innovations from Polio Eradication (STRIPE) project, which conducted an online survey and hosted key informant interviews with individuals who had been engaged with the GPEI program from 1988 onwards for at least 12 or more continuous months. An analysis of data limited to individuals (32%, N = 3659) who were primarily involved in CE activities revealed that around 24% were front-line healthcare workers, 21% were supervisors and 8% were surveillance officers. CE activities mainly focused on building trust within the communities, addressing misinformation, myths and fears around vaccinations, mobilization to reach high-risk or hard to reach populations, as well as building ownership and buy in from the communities. The strength of the implemental process of a program (38.7%) was among the key drivers of success, coupled with personal beliefs and characteristics of the implementers (25.3%). Social, political, and financial forces received mixed opinions as to their importance, depending on the stage of execution and readiness of the communities to accept the programs. Lessons learnt from the GPEI program provide tried and tested best practices and evidence for strategies that would work in diverse backgrounds with some customization to suit the needs of the situation.

## Introduction

Community engagement, a blend of concepts and approaches based on mutual respect and inclusion to achieve shared goals and visions among diverse actors at the community level, is a complex strategy. Community engagement (CE) has been variously framed as community involvement, community consultation, community participation, and community partnership in public health research, and holds different meaning for different stakeholders [1, 2]. CE has historically been an important component of public health research and program implementation, especially in low- and middle-income countries, and has been scrutinized more recently due to ethical concerns regarding how it is implemented within vulnerable and disadvantaged groups of the society [3, 4]. Over the years, there has been a gradual shift in the emphasis in CE priorities, priorities initially focused on involvement, ownership and consultation with relevant communities or end-users in research, and later morphed into partnership in research and program implementation processes and advocating for policy recommendations [5]. The latter emphasis on partnership in research and implementation processes, and in advocating for policy recommendations has been conceptualized as community based participatory research, which aims to improve acceptance and reduce disparities of public health research activities and benefits in the involved communities [6–8].

The advances in technology are also changing the way public health researchers are engaging with communities in data collection as well as implementation and roll out of health interventions, and there are unanswered questions in how these interactions are mutually and ethically beneficial for all those involved [9]. However, rigorous evaluation of the application of community engagement has been seen only in a few select areas of public health research such as women’s health and climate change [10]. Most of these evaluations have focused on the impact of community engagement from the beneficiaries’ point of view. The implementation of community engagement as a stand-alone objective or in combination with others has not been studied with the same fervor from the implementer’s perspective, i.e., individuals and actors who use community engagement as a strategy to facilitate the delivery of health interventions, and these individuals can be anyone in the chain of processes that starts with the development of the intervention to execution, such as researchers, front line workers, technicians, government employees, non-governmental employees, and policy advocates among others [4, 11, 12].

The Global Polio Eradication Initiative (GPEI), i.e., World Health Organization (WHO), Center for Disease Control and Prevention (CDC), United Nations Children’s Fund (UNICEF), Bill and Melinda Gates Foundation (BMGF) and Rotary International, aimed at eradicating poliomyelitis, is one of the largest public health programs ever undertaken globally. Over the years, the GPEI has implemented and revamped various strategies, including CE, to increase immunization coverage for polio vaccines, and has worked to reduce the burden of polio, improved surveillance systems, and expanded health access to vulnerable and hard-to-reach populations [13, 14]. Given the global reach of GPEI and its implementation activities in diverse communities, the tacit knowledge [i.e., experiential knowledge that may not have been previously documented, widely published or disseminated) gained from the individuals who were part of the eradication program and involved with the objective of community engagement would provide insights and knowledge on how CE works in practice from the implementers’ perspective, facilitate the success of other global health initiatives, and produce generalizable theories on the dynamic interplays between public health research and practice and communities [15]. This paper highlights the experiences of individuals who worked with the GPEI from 1988 onwards, with special focus on contributors and challenges to the implementation of the GPEI program’s community engagement initiatives from an implementers’ perspective.

## Methods

The study’s analysis was informed by data collected from the Synthesis and Translation of Research and Innovations from Polio Eradication (STRIPE) project, which conducted an online survey to capture perspectives of individuals involved in GPEI across diverse contexts, in seven focus countries, Afghanistan, Bangladesh, the Democratic Republic of Congo (DRC), Ethiopia, India, Indonesia and Nigeria, that represent different geographical regions and income classifications, and that had high activity in the polio eradication campaign [16]. The survey is part of the larger STRIPE initiative which aims to map, package, and disseminate knowledge from polio eradication initiatives in the form of academic and training programs for health audiences to inform the implementation of similar programs globally [16].

The sample for the STRIPE polio survey comprised of those individuals 18 years and above who were directly involved in the implementation of the eradication program under GPEI from 1988 onwards for at least 12 or more continuous months at the global level, and in the seven countries. This sample was taken from an enumerated source population of 193,096 individuals that fit the sampling criteria, and the response rates varied from country to country, ranging from 7% in Ethiopia to 35% in the Democratic Republic of Congo [17]. Detailed sampling methodology for the survey has been captured elsewhere [18]. The web-based online survey, developed on Qualtrics, was informed by the Consolidated Framework for Implementation Research, the Organizational Social Context Framework, and the socioecological model [19–21]. The survey covered key constructs describing the internal and external contexts for implementation, implementation strategies, intended as well as unintended consequences and other descriptive characteristics such as organization, level of engagement and demographic information related to the respondents [20–22]. The survey was self-administered and was implemented using the Qualtrics electronic data platform in seven languages–Baha-Indonesia, Bangla, Dari, French, Hindi, Spanish and Urdu. In some locations where electronic data collection was not possible, in person or telephone-based interviews were conducted in the local language and later translated into the English language to complete the survey. More details of the implementation of the survey and the larger project have been described elsewhere [17].

For the purposes of this study, the analysis was restricted to individuals who selected community engagement as one of the three goals they spent most of their time on within the GPEI activities. Community engagement was operationally defined as organized efforts to improve demand and uptake of polio vaccines via improved campaign outreaches, behavioral change interventions, advocacy with community leaders, and other strategies to counteract misinformation and trust among discrete populations. All analyses were conducted using STATA I/C (version 15) [23]. A sub-set of the sample population that responded to the survey were also engaged in key informant interviews to further explore the challenges to implementation of the GPEI programs. The interview guides were conceptually aligned with the socio-ecological framework and data gathered was analyzed using Dedoose (version 8.2.31) [17, 24]. Data were coded and analyzed by two reviewers from two different countries, and the data was validated post-analysis including cross checking with key respondent findings [17].

Descriptive analyses were used to report frequency and percentages of those individuals involved in community engagement across various demographic characteristics (organization type, role, years of engagement, level of engagement) and perceptions of those individuals towards the facilitators and barriers to the implementation of community engagement activities. Free text survey responses on successes and barriers to implementation of CE, as well unintended consequences, and solutions, were extracted into an excel sheet and coded manually using focused coding techniques [25]. The codes were thematically reviewed, analyzed, and categorized into conceptually similar categories. The categories were further aggregated into overarching themes [25]. Additionally, the quotes and themes generated from the key informant interviews were reviewed to extract quotes that were thematically aligned with the themes generated from the survey on CE. The quotes were then incorporated into the current analysis to provide contextual and circumstantial understanding of the barriers and facilitators to the implementation of community engagement activities for the GPEI programs.

The study was approved by the Institutional Review Board of the Johns Hopkins Bloomberg School of Public health and was deemed to be “non-human subjects research” as no personal health information or any identifiers were collected from the participants (IRB No: 00008721). Informed consent was obtained from all research participants. Survey participants were provided a written consent statement prior to accessing the survey. Oral consent was obtained from KII participants and from phone-based survey participants.

## Results

Of the 3,659 individuals who completed the STRIPE survey and had at least one year of experience working on GPEI activities, 1,105 (32.1%) worked mainly on the polio program goal of community engagement (CE) and were part of this analysis. The average length of time working in community engagement was 8.7 years (±6.5); 312 (31.1%) respondents had been working for 10 to 20 years, 316 (31.5%) for 5 to 9 years, and 309 (30%) for 1 to 4 years. A total of 65 (6.5%) individuals had more than 2 decades of experience being involved with CE (Table 1). The contributions of these individuals occurred at various levels–global, national, state, district, and sub-district–a total of 686 (62.2%) worked at only one level while 419 (37.9%) worked at more than one level at the same time. Of all the respondents, 278 (25.2%) worked at the national level, 164 (14.8%) at the state level, and almost half (48.5%) worked at the district/sub-district level.

**Table 1:** Characteristics of respondents involved in community engagement.

	Survey respondents, (N = 1105)	
	n	%
**Levels worked** ^**1**^		
Global	164	14.84
National	278	25.16
State	416	37.65
District/ Sub-district	1118	48.48
**Organizational representation** ^**1**^		
GPEI partners^4^	680	36.34
Governments	1103	38.25
Implementing organizations	529	18.34
Research organizations	43	1.49
Others	161	5.58
**Primary roles** ^**1**^		
Advisory capacity	81	7.33
Strategy	48	4.35
Manager/ officer/ supervisor	509	46.02
Front-line health worker	266	24.07
Others^2^	198	17.9
**Years of experience** ^3^		
1 to 4 years	309	30.84
5 to 9 years	316	31.54
10 to 14 years	208	20.76
15 to 19 years	104	10.38
20 years and more	65	6.49

^**1**^ For the levels respondents worked, their organizational representation and longest primary roles held over the 1988–2019 period, the respondents were able to select multiple responses; the sum of responses (n) under each of these characteristics is greater than the number of respondents who selected community engagement as their primary polio program goal (N = 1105)

^2^ policy makers, researchers, communications and coordination, technical support, consultants, volunteers

^3^ 9.32% observations missing

^4^ World Health Organization (WHO), Center for Disease Control and Prevention (CDC), United Nations Children’s Fund (UNICEF), Bill and Melinda Gates Foundation (BMGF) and Rotary International.

There was no significant association between years of experience and multiple levels of engagement. Of those who had more than 2 decades of experience, two-third of these individuals had worked only at one level. Working at district and sub-district levels was also associated with more years of engagement. Similarly, the years of engagement were also significantly associated with those who worked as front-line health care workers, Expanded Program on Immunization (EPI) managers, and those who were at an advisory capacity at the national level. These were significant at a p value of 0.001 or less.

More than 24% of the individuals engaged in community engagement were front-line health workers (n = 266) followed by supervisors (235, 21.3%) and surveillance officers (88, 7.9%). 129 (11.7%) individuals were involved at the global, national, or sub-national level in an advisory or strategy development capacity in assisting with the objective of community engagement.

### Community engagement activities

Based on the free text responses from the survey, the self-reported community engagement activities covered diverse strategies, including strategies to mobilize community members for action, engage prominent figures and faith leaders, seek participation of marginalized populations and challenge stereotypes and social hierarchies among others.

An overwhelming majority of the community engagement activities self-reported by the respondents fit well within the scope of social mobilization e.g., engaging champions and leaders from the community to spearhead the messaging around the program or involving faith leaders and religious structures as safe places to discuss and troubleshoot concerns about program implementation. Other strategies such as house to house promotions via pamphlets and sharing outcomes of the program through trusted members of the community fall within the scope of community action. (Table 2)

**Table 2:** Community engagement activities in GPEI as reported by respondents.

Goals of community engagement activities in GPEI	Example of strategies
Trust building	Engaging local celebrities, faith leaders Engaging local political leaders Use of religious and traditional structures and community gatekeepers to influence behavioral change Recruiting community health workers (e.g., anganwadi workers in India) from within the communities for ease of communication Engaging females in the communities as vaccinators for access to households Engaging youth groups/ students/ teachers for community mobilization House to house promotions Community group formations and education on diagnosis Encouraging community ownership of implementation process
Reaching high-risk/ hard to reach populations	Engaging military for access, immunization, and surveillance Multi-agency collaboration (governmental and non-governmental) to solve logistical challenges Utilization of high-movement avenues to discuss and resolve concerns House to house promotions for information sharing
Addressing misinformation and trust	Involving community leadership, faith leadership, opinion leaders and other active groups in the planning and execution of in-ground activities Engaging media at all levels from national to local to provide accurate information about a program Mandating government health bodies to provide accurate information regarding a health program
Building sustainable community-based local capacity	Involving community members in training for door-to-door vaccination drives and health messaging

### Facilitators and barriers to community engagement activities

Based on the Consolidated Framework for Implementation Research that guided the development of the survey tool, the facilitators and barriers to community engagement activities were segregated as internally or externally facing. (Table 3) Internally facing characteristics that were drivers of success mainly contributed to the implementation process of the program (38.7%) and personal characteristics, beliefs and commitment of the individuals directly involved in program implementation (25.3%). Further, respondents also identified social context (50.9%), political climate (23.4%), and financial forces (10.9%) external to their implementing organizations as factors that led to the overall success of the program.

**Table 3:** Facilitators and barriers to community engagement activities.

Contributors to success		Survey respondents (N = 1105)	
		N	%
**Internal** ^**1**^	Definitions		
Individual characteristics	Factors related to attitude, own beliefs, training, and skills to conduct community engagement activities as well as organizations’ perceptions	280	25.34
Organizational characteristics	Factors related to organization supporting the polio eradication program	143	12.94
GPEI program characteristics	Activities used towards eradicating polio, including technologies	148	13.39
Process of conducting activities	Activities implemented, including the planning, execution strategies, reflection and evaluation of activities, or adjustments made to the plan	428	38.73
**External** ^2^			
Political environment	Lawmaker support, political climate accepting of polio eradication activities, and political structure conducive to coordinated action	259	23.44
Economic environment	Sufficient revenue sources/base to fund activities and/or maintain system developments	120	10.86
Social environment	Social norms around immunization, accepting communities in which polio eradication activities were implemented	563	50.95
Technological environment	Infrastructure or technological advances outside of the organization	29	2.62
Others		23	2.08

^1^ 9.59% missing values

^2^ 10.05% missing values

### Contextual background of the internal and external factors

Of all the respondents who participated in the online survey, 194 individuals also participated in the key informant interviews. Data from respondents who were engaged in community engagement activities were included in the below thematic analysis [17].

Among the respondents who were engaged in community engagement activities, 38.7% reported that the implementation process (including how the activities were planned, executed, and evaluated) played a key role in facilitating engagement with the community (S1 Table). Regular internal discussions, re-strategizing to meet the changing needs and demands, engaging with new collaborators such as consulting firms to support changes in governance and management structures, and cohesive workflow with government and other stakeholders were some unique processes mentioned by interview participants.

*There were instances in Northern Nigeria that then very much affected the whole Central Africa area zone in terms of the ability to carry out some of the vaccination activities going all the way back to the early 2000s to 2004*, *2005 and resulted in outbreaks following that*. *I think these types of challenges led to the program having to really modify some of its strategies specifically related to communication and communicating with not only influential stakeholders but also mothers and caretakers and all the way down at the community level*, *things like that have in a sense the adversity that’s kind of presented to the program*, *it’s forced the program to grow and to change*–*National level*, *Nigeria*

Further, the implementation process that facilitated success of CE activities involve discussions, real-time learning and feedback, task shifting and sharing resources among collaborators, as highlighted below:

*…we learned the importance of working closely with other partners. We may have similar program. To save resources we will share tasks to reduce duplication of activities and wastage of resources. One partner takes some tasks, and the other takes another one. I remember when they asked us to support review meeting. We then ask another partner who is working on the same area. We discuss and one of us paid for the per diem and the other partner paid for the rent. This helps you to broaden your imagination in doing a good work–sub-national level governmental organization.–Sub-national level, Ethiopia*

The process of implementation was however also a significant challenge for around 23.9% of the survey respondents, 46.6% of whom believed that difficulty in engaging key individuals, stakeholders, and other appropriate organizations in implementing the polio program was a major challenge. Around 33% (of the 23.9%) suggested that the perceived complexity of the polio eradication program–scope and duration of implementation, multifaceted approach, lack of internal communication–were challenges to achieve the objective of community engagement.

In addition, the personal characteristics of the individuals implementing the CE activities within an implementing organization (such as work ethics, commitment to the cause) facilitated the success of the community engagement activities, according to 25.3% of the respondents. In situations where positive individual characteristics worked synergistically with strong leadership, significant gains were achieved with the implementation of the CE activities as mentioned below-

*It’s a combination of the design*, *the quality of the work*, *the commitment of these local staff*, *whether they were unpaid community-level workers*, *to the full-time staff at the secretariat level*, *and a lot of the people working in the CORE Group member project offices in these countries …*. *Again*, *some of that is the leadership skills of these national directors and the staff that they surrounded themselves with*, *but there was a real commitment to this–*Global level

On the contrary, 18.5% of the surveyed individuals experienced significant challenges due to the personal characteristics of individuals working in their organizations. Those respondents (68.6% (n = 140) who encountered challenges with individuals with negative attitude towards the polio eradication activities reported that the lack of commitment towards the program was a major hindrance. Limited resources for use and incentives, lack of connection with leadership, below par compensation for work put towards the engagement activities, and poorly trained workforce impacted the motivation and workflow of health workers involved in CE activities, and the actual delivery of those activities, as mentioned below. In some settings, these factors were exaggerated by the mere lack of functioning and sustainable health care systems to support the GPEI activities. It is however important to note that the perceived lack of health worker motivation derived from a complex set of factors that were not just restricted to CE activities but applied across the other GPEI programmatic objectives.

*… what is ticking is that there is no real follow-up, especially of the hierarchy… There are tools they must give us to function better, the central office is also limited. Let us have training to have knowledge not acquired on the school bench. Let them also equip structures starting from the central office, it will work better. Let them fix the pay especially for the community relays because it is a difficult job, it is they who work in the field, it is they too who give us the data that we take to the hierarchy. They are threatened. The hierarchy benefits from it but those who do the work on the ground do not benefit from it. During the campaign, they [community relays] say they waste their time all day. They are also parents and at the end of 3 days they are given only $15. And while they are working for the country, their children are suffering—sub-district level IT technician*

The communities themselves (external environment) were a barrier to enacting community engagement activities, as reported by 48.9% of the individuals. The perception of communities towards the polio program and related field activities was a major challenge (50.8%). Some communities were resistant or non-accepting of the polio vaccination, which translated to push back and suspicion of related community engagement activities, especially in low-resource communities. Socioeconomic inequities (poverty, limited economic opportunities, and low education status) combined with personal religious beliefs, disinformation and misinformation about vaccinations, and the influence from spiritual healers and alternate medicine practitioners negatively impacted the uptake of CE activities in some of these low-resource communities. Furthermore, an assumption that literate folks in these communities were aware of the importance of vaccinations (and were therefore targeted by CE activities) further contributed to challenges in implementing the CE activities overall -

*…. awareness is not as intensive as it should, whether at the local level or at the higher, at the let me say the literate level because you tend to do the awareness with the illiterate more than the literate. … You may think they are literate, but they still need to be told and they still need to be educated, enlightened … because usually when we are giving non-compliance it is usually more of the literate people–sub-national level, Nigeria*

The political climate acted as both a facilitator (23.4%) and a barrier (24.6%) to the implementation of various community engagement activities. Support and acceptance of the program objectives from national and sub-national political players as well as buy-in from local governments made it easier for the front-line workers and others to implement community engagement activities with ease.

On the contrary, some teams faced resistance from policy makers making it difficult to engage with communities. In some locations, the evolving and dynamic state of the political structure was not conducive to coordinated action by implementers. These also led to security concerns due to armed conflict and migration of communities. The general mistrust as well as poor utilization of available resources by government agencies were some politically inclined issues that created challenges in community engagement activities. Local teams innovated to get around the security risks by collaborating with the military in some regions such as Afghanistan and Nigeria -

*… so, in Afghanistan it is such a complex situation that the basic principles that we set for program was the program neutrality. We just wanted to have the program as much neutral as possible without any visible engagement of any parties of conflict. Then the second basic principle that we set is the safety and security of the vaccinators, because this is also important, that those people were going and serving those areas—sub-national level, Afghanistan*

… in some of the areas of Nigeria the innovation which we call RIC, Reaching Inaccessible Child—it is an innovation where actually some of those areas which are already identified, their vaccination and even sometimes surveillance is done by military personnel all by themselves–sub-national level, Nigeria

The economic climate also played a role in hindering the respondents (29.4%) from implementing community engagement activities successfully. Lack of funds for procurement of supplies, poor remuneration and insufficient incentives or compensation demotivated front-line workers in conducting the engagement activities to their fullest extent.

It was hinted by a few that improved and targeted dissemination and distribution of the messaging around the polio campaign would lead to more open engagement with communities. Respondents mobilized the community by involving local celebrities, faith and religious leaders and traditional healers to provide crafted messaging to raise awareness about the campaign, as suggested below:

*… people have concerns about vaccines, and they are refusing vaccines. This is a negative point…we had to talk to them several times and managed to convince them. We involved community in the discussion with elders and their representatives. We also involved community volunteers. We have 3 to 4 committees of volunteers in every district. They have specific days for refusal families and target them with the collaboration of community elders and volunteers. We convince them and give vaccine for the children–Sub-national level, Afghanistan*

*We also trained Malik (local community elders) and provided messages through TVs and mosques. We also trained staff of clinics such as CHWs [community health workers] to do community awareness. We have resolved this issue. The other big thing we have done is the recruitment of local people for teams and this has been instrumental in tackling this issue. Team members are recommended by local elders, so they are from the community; they have relatives there and are trusted by families–Consulting firm, Afghanistan*

Some respondents suggested engagement of community members in the planning and execution of activities on the ground to provide the communities with ownership and control of the campaign.

### Collateral benefits of community engagement activities

In some countries, like Afghanistan, where experiential evidence from the field had shown that social norms were against women working as vaccinators, especially in rural areas, female members of the community that were employed as front-line workers and vaccinators were given access to households and could make sure all children were getting immunized while gaining employment. As shared by a respondent, “*positive changes this (program) has brought to women in spite of some terrible acts of violence*.”

Due to the interaction of local government agencies with the communities during awareness campaigns and door-to-door visits by front-line health care workers, surveillance for other communicable and non-communicable diseases improved, setting a system for obligatory notification of causes other than polio. Collaborations were seen to organize health camps in underserved areas, promote hand washing, importance of nutrition and other related topics.

*From the health system, obviously, the people in society become [more] aware of prevention. Because [there is better access] to other vaccines for example Measles, Rubella, Hepatitis, Hepatitis A, Influenza. Our people have become aware of immunization…. In some locations, health facilities teamed up to conduct additional services such as malnutrition screening, latrine coverage and bed net checking for malaria–sub-national level, Indonesia*

Additionally, health education and training programs were conducted for field staff to provide them with the knowledge required to improve communication with communities. These programs also supported capacity building of the staff in other aspects of the program that could be translated to other service delivery programs. The development of infrastructure to support the polio program also provided the strength for localities to address other health-related programs.

*Not only did the health service delivery strengthen, but the training programs also provided to sustain the polio program produced trained workforce in the fields of surveillance, monitoring and evaluation, communication, logistics management, partnership building, networking, coordination, innovation, micro planning, multi culture working approach, observation, and date analysis among others… This not only reduced mistrust towards the government but also increased awareness of vaccinations among communities and their confidence in health care providers and caregivers*.

*In hard-to-reach areas, the polio program supported the delivery of other basic clinical and curative services by providing a channel for others to engage in interdisciplinary and intersectoral activities–sub-national level implementing organization- district level, Nigeria*

The awareness programs and health education activities conducted to raise awareness within communities was found to have developed interest in youth to focus on science and biology. The mobilization programs also improved literacy of local communities in health services delivery, access, and basic hygiene such as maintaining clean environment and living healthy life.

## Discussion

The findings from this study show that individuals involved in community engagement activities faced multiple barriers in the implementation of specific activities geared towards achieving the objectives of the Global Polio Eradication Initiative. Community engagement activities were mainly targeted to increase vaccine acceptance and improve awareness and trust within communities that would assist in achieving the overall objective on increasing polio vaccination coverage. Over the years of implementation, the GPEI program transitioned to engage more with communities via its community engagement strategies, which improved its understanding of the importance of buy-in from end users. Based on the country context, community-based surveillance, campaign monitoring, cross-border initiatives and presence of community mobilizers were among a few innovative strategies that were implemented to improve vaccination uptake in communities [26] Past reporting on community engagement activities by the CORE Group highlight that need for community participation as community support or lack thereof can help identify targeted, creative strategies for specific problems, for example when communities showed distrust in the polio vaccine. Engaging with the end users of the program provides the implementers with opportunities to learn about the communities they are serving and conduct culturally adept activities [27, 28].

Conflict was a deterrent in the initial efforts at community engagement activities in different settings as implementation processes were not able to embrace the contextual and cultural nuances of the community. There was backlash in the form of distrust and struggle in carrying out planned activities, as well as violence towards the front-line health care workers and vaccinators, who were primarily part of the same communities [12, 29, 30]. However, by working closely with local partners with deep understanding of the communities, political and influential support and timely adjustments to the program, community engagement activities were able to go a long way in creating awareness and importance of interventions [31–34]. Reports from Ethiopia share examples of community volunteers being selected by the community members, which solidified credibility, trust and representation of community needs [35]. India’s experience with mistrust among communities about the use and implications of the polio vaccine and strategies developed by the program, such as engaging school children and teachers as immunication champions or mobilizing community members to be volunteer influencers are some examples of leveraging the communities to achieve success for a program [36].

Interactions with fellow workers as well as communities defined the trajectory of the program, leading to differing perceptions of success. The process of implementation was a big facilitator in making the community engagement efforts a success to support the eradication program [14]. The planning and execution of community engagement activities as well as feedback and reflection on those activities within the organization and other stakeholders improved the delivery of the engagement activities [14]. The implementation process when not clearly defined and aligned with the human resources within an organization turned out to be an impediment to smooth implementation of the activities. Experiences from India suggest that emphasis needs to be placed on strong partnerships and coordination mechanisms among internal and external stakeholders for programs to be delivered in communities with success [37].

The external environment–a mix of social, political, economic, and technological issues–was highlighted as a key barrier to engagement with communities to deliver the intervention. The social structure has played an important role in supporting the implementation of maternal and child health programs, HIV/AIDS prevention campaigns, nutrition programs, among others [11, 38–40]. Not only that, prior evaluations of the GPEI efforts in different countries have shown that buy-in from communities via community engagement activities such as awareness campaigns, information and education sessions, door-to-door messaging, cross-border initiatives, among others is important for parents to maintain compliance to vaccinations for their children [41]. The GPEI efforts have also set up operating systems within communities to distribute bed nets for malaria prevention in Nigeria or provide deworming medication for Guinea worms in Ethiopia as a way to establish a footing and build trust with those communities [42, 43]. Additionally, strategies developed and improved as part of the GPEI efforts have the potential to improve maternal and child health across other communities, hard to reach, marginalized populations across the globe [44].

The feedback from the individuals involved in engaging with communities reiterated the importance of collaborating with local governments and leaders, high profile residents and religious influencers in crafting the implementation strategy of the program. Their support in raising awareness and helping fight rumors and taboo around polio and vaccinations in general was necessary to have more communities comply with the vaccination program [45, 46]. In countries like India and Bangladesh, the Rotary Clubs have been core collaborators of the polio initiative in utilizing their network of Rotarians and other resources to conduct grassroots activities [47]. Such collaborations have historically supported the implementation and uptake of other programs as well, not only limited to communicable and non-communicable diseases but violence, suicide, and mental health related stigmas [48–51]. While collaborations and strategies are required to successfully achieve community engagement efforts, it is imperative to have organization management, coordination, supervision, monitoring and evaluation and research to realize utmost benefits of programs within communities.

While many studies have highlighted the lack of knowledge and awareness among communities to comply with vaccination programs, this study showed that the individual characteristic of employees, their attitude towards the programs as well their inherent knowledge and beliefs towards vaccinations in general had an impact on how they interacted with the communities in delivering the vaccinations [41, 52]. Per diems for front-line health workers, lack of security for field staff, and poor remuneration for staff were some additional individual-based factors that lead to demotivation and loyalty towards the implementation of respective responsibilities [53, 54].

It is important to reflect on what the findings from this study mean against the backdrop of recent rise in the outbreaks of vaccine-derived polio virus (cVDPV) globally. For instance, cases of cVDPV type 2 (cVDPV2) ranged from 1 (in Ethiopia and Indonesia) to 255 (Democratic Republic of Congo) in 2022 across the seven countries considered in this study [55]. This rise calls for a sustained community engagement effort, especially around sustaining community participation in identifying bottlenecks to vaccine uptake broadly and identifying strategies for addressing these challenges as they arise–not only in these seven countries, but other countries globally.

The study does have some limitations. First, while the survey tool was created within a particular framework and clear definitions were provided; responses and recall biases might have been introduced in the data as responders may not have addressed the questions in the same frame. Therefore, it is important to keep in mind how some of the variability in the responders’ perceptions may have influenced our conclusions while extrapolating these findings across other service delivery programs. Second, the study reflected its findings with a focus on the internal and external factors that lead to the success of the CE activities–detailed analyses are required to delineate the perceptions of respondents across specific roles towards CE activities. Third, we did not have information on the specific time periods when a respondent was engaged in CE activities to be able to capture change in perception to CE over the years. However, we know that the bulk of our respondents (>60%) had 10 years’ experience or less, and our findings may be influenced by recency bias.

### Conclusion

Community engagement has become an integral component of program implementation and plays a unique and important role in the success of a program. Understanding and executing strategies to engage communities in a program process is critical to recognize the nuances that can streamline the uptake of an evidence-based program within communities and fulfil the objective of the program. The nuances recognized from a program as large scale as the Global Polio Eradication Initiative can support and preempt the thinking and strategizing around barriers to community engagement for other similar programs that aim to reduce mortality and morbidity within communities due to communicable and non-communicable diseases. Lessons learnt from this initiative provide tried and tested best practices and evidence for strategies that would work in diverse backgrounds with some customization to suit the needs of the situation.

## Supporting information

S1 Table Barriers to success of community engagement activities. (DOCX)

S1 Questionnaire Inclusivity in global research questionnaire. (DOCX)
