# Supplementary material for: Facilitators and barriers to community engagement in the Global Polio Eradication Initiative–A mixed methods study
Source: PLOS Glob Public Health. 2023 Apr 7;3(4):e0001643. doi: 10.1371/journal.pgph.0001643 (PMC10081736; doi:10.1371/journal.pgph.0001643)
Supplement: S1 Table — (DOCX) [file pgph.0001643.s001.docx]

S1 Table: Barriers to success of Community engagement activities

| **CFIR Domain** | **Barrier Definition** | **Survey Respondents**  **(N =1105)**  **n1 (% of N) ***  n2 (% of n1) ** |
| --- | --- | --- |
| **Characteristics of individuals** | **Characteristics of individuals within an organization involved in polio eradication activities** | **204, 18.46** |
| Knowledge | Knowledge and beliefs about the activity - individuals did not have positive attitude toward the polio program, were unfamiliar with facts, truths and principles related to the intervention | 140, 68.63 |
| Stage of Change | How likely (or not) the individual is to provide skilled, enthusiastic and sustained support of the program throughout the different stages of implementation | 55, 26.96 |
| Perception of organization | Poor perception of the organization and degree of commitment to the organization | 48, 23.53 |
| Self-efficacy | Lack of belief in one’s own abilities to execute required courses of action | 38, 18.63 |
| Other | Challenges related to physical and human geography | 13, 6.37 |
| **Process of activities** | **How activities were implemented** | **264, 23.89** |
| Executing | Failing to carry out activities according to plan | 135, 51.14 |
| Engaging | Difficulty attracting and involving appropriate stakeholders in implementation | 123, 46.59 |
| Reflecting & evaluating | Difficulty monitoring program progress and quality, including lack of regular debriefing about progress and experience | 89, 33.71 |
| Planning | Implementation schemes/methods in advance not planned, or poor quality of such methods | 82, 31.06 |
| **Organizational characteristics** | **Factors related to the organization(s) supporting implementation** | **121, 10.95** |
| Structure | The age, social architecture, and size of an organization led to challenges | 25, 20.66 |
| Networks | The nature and quality of formal and informal communication within an organization led to challenges | 48, 39.67 |
| Culture | The norms, values, and operating assumptions of an organization led to challenges | 46, 38.02 |
| Implementation Climate | Limited capacity for change, the receptivity of the team to the proposed intervention, the relative priority of project, organizational goals, incentive and rewards, etc. led to challenges | 40, 33.06 |
| Implementation Readiness | Lack of leadership engagement, limited available resources and poor access to knowledge and information led to challenges | 55, 45.45 |
| **External Factors** | **Political, economic, social, technological, legal, and other environmental factors** | **541, 48.96** |
| Social | Communities are non-accepting and/or resistant to the intervention | 275, 50.83 |
| Economic | Insufficient revenue sources | 159, 29.39 |
| Political | Policymaker disinterest or resistance, limited windows of opportunity within the political climate, political structure non-conducive to coordinated action | 133, 24.58 |
| Technological | Slow or limited advances of technologies used in implementing program activities | 61, 11.28 |
| Other | Challenges related to physical and human geography | 112, 20.70 |
| **Program characteristics** | **Activities conducted to enable implementation, including technologies adopted** | **103, 9.32** |
| Intervention Source | Perception of whether the intervention was developed internally or externally led to challenges | 31, 30.10 |
| Evidence | Perception of the quality and validity of the evidence did not support belief that the intervention would have the desired outcomes | 35, 33.98 |
| Relative Advantage | Perception that there was another, better approach | 25, 24.27 |
| Adaptability | The activity was not adapted, tailored or refined to meet local needs | 45, 43.69 |
| Trialability | No ability to test on a small scale and reverse course if warranted | 13, 12.62 |
| Complexity | Perceived difficulty of implementation reflected by its duration, scope, radicalness, disruptiveness, centrality, intricacy, and number of steps required | 34, 33.01 |
| Design Quality & Packaging | Difficulty arising from how the intervention is bundled, presented, and assembled | 23, 22.33 |
| Cost | Cost of intervention and its implementation, including investment, supply, and opportunity costs | 32, 31.07 |

*Each respondent was allowed to choose all relevant domains that contributed as barriers to polio program goals. Hence, the sum of all responses, n1 is greater than sample size for all survey respondents who contributed to community engagement (1105).

**Within each domain, respondents were similarly allowed to choose all relevant categories that contributed as barriers to polio program goals, e.g., for the external factor domain, each respondent selected multiple categories under that domain such that the sum of all category-specific responses (n2) is greater than n1 for that domain.
